# Astrocytes reassessment - an evolving concept part one: embryology, biology, morphology and reactivity

**DOI:** 10.1186/2049-9256-1-18

**Published:** 2013-10-24

**Authors:** Alina Simona Şovrea, Adina Bianca Boşca

**Affiliations:** Discipline of Histology, Department of Morphological Sciences, Iuliu Haţieganu University of Medicine and Pharmacy, Cluj-Napoca, Romania

**Keywords:** Astrocytes, Reactive astrogliosis, Molecular mechanisms, Therapeutic targets

## Abstract

The goal of this review is to integrate - in its two parts - the considerable amount of information that has accumulated during these recent years over the morphology, biology and functions of astrocytes - first part - and to illustrate the active role of these cells in pathophysiological processes implicated in various psychiatric and neurologic disorders – second part.

## Introduction

Increasing research interest aroused by astrocytes over the past few years led to a dramatic evolution of the concept regarding their structure and function. Ubiquitously present in all regions of the central nervous system (CNS), astrocytes are specialized glial cells, providing structural and functional support for neurons.

Although considered for more than 100 years as a homogenous cell population, it is known today that glia encompasses various morphological entities that coexist; each of these populations are characterized by a particular molecular signature and specific functions related to their microenvironment. Moreover, dysfunctions of astrocytes might contribute to CNS pathological remodelling and disease [1].

## Review

### Short history

The concept of neuroglia, introduced by Rudolf Virchow in 1858, described a connective substance of the brain, represented most likely by “fibers and intercellular masses”. Otto Deiters, a German scientist, was the first who, in the second half of the 19^th^ century, drew the astrocytes as stellate cells; later, Jacob Henle and Friedrich Merkel observed the network formed by the astrocytes processes within the grey matter [2]. Yet it was Camillo Golgi (1872) the first who detailed and described the morphology of glial cells by using the silver-chromate technique (a black staining reaction); he observed that some glial cells (known today as protoplasmic astrocytes) displayed endfeet on their processes, attached to the blood vessels. His theory postulated that there was a link between the morphology and function of astrocytes in the CNS; regarded as the “glue” of the brain, glial cells established an interconnection between vessels and parenchyma, therefore being responsible for metabolic exchanges. In 1893 Michael von Lenhossek contrived the term “astrocyte” that illustrated the morphology of these cells. The origin of this term arouse from a combination of the latin word for stars, *astra,* with the word for cell, *cyte*, thus a star-shaped cell [3]. Astrocytes were further classified into protoplasmic (found in the grey matter) and fibrous (within the white matter) [2–4].

At the beginning of the 20th century the morphological heterogeneity of the CNS glia was definitely set. However, only when Santiago Ramόn y Cajal (1913) has developed the gold chloride-sublimate staining technique, the first specific stain for astrocytes, this diversity was acknowledged. Cajal is considered the promoter of the future stem properties of neuroglia since, using this method, he proved that astrocytes originate from radial glia and undergo cell division in the adult brain. Numerous functions of astrocytes (e.g. neuronal nutrition and metabolism, nervous tissue homeostasis, brain cytoarchitecture, glial scar formation) were further determined, relying on Cajal’s histological research, rendering astrocytes essential brain “homeostatic cells” either in normal or pathologic conditions [5].

Yet, the gains regarding the functions of astrocytes were shadowed by the lack of adequate techniques that could have promoted them, versus neurons of which value was overstated by the neuronal doctrine [2].

### Phylogenetic evolution

From the phylogenetical point of view, the organization of a centralized nervous system was marked by the appearance of astrocytes [5].

An interesting aspect is the constant augmentation the astrocytes/neurons ratio that parallelized the evolution of the brain (about 0.16 in nematodes to 0.33 in rodents, and reaching up to 1.65 astrocytes per neuron in the human cortex) [6]. It is considered that, in the human brain, to each neuron correspond 10 glial cells. In smaller creatures’ brain, the number of glial cells corresponding to a neuron is significantly reduced [7].

The primordial astrocytes performed a wide range of functions in the development of the nervous system. In nematodes, the astrocytes are not only involved in neuronal development, but also enable the sensory functions [5]. Moreover, the astrocytes’ performances improve with the evolutionary stages. For example, in arthropods glial cells fulfill an additional role, organizing the neurons in functional definite nervous centers [5]. In crustaceans, insects and cephalopods, even in some vertebrates (sharks), the astrocytes form the blood-brain barrier (BBB) or the hemolymph-brain barrier (HBB) isolating the nervous tissue from the rest of the body [5]. Primordial astrocytes also envelop the axons therefore being the predecessors of the myelin forming cells; the astroglial sheath of the axons improves the propagation of the action potential [5]. In higher vertebrates, astrocytes’ role in maintaining the BBB function is completed by the endothelial cells. Besides, in this stage of evolution, astrocytes specialize for the defensive function [5]. In humans, astrocytes achieve their greatest morphologic and functional complexity. For example, neocortex humans astrocytes compared to those of rodents, are 2.5 times larger, their processes are 10 times more numerous and they display particular histological features; the action potential velocity is also 4 times higher [7].

### Stem cells and astrocytes differentiation

Initially astrocytes were identified due to their star-shaped morphology and presence of the glial fibrils. Nowadays these features are almost outdated.

The diversity of astrocytes is justified by two main factors: the heterogeneity of glial precursors and the various pathways of specific differentiation, both being influenced by the extracellular environment. Recent *in vitro* studies reported that growth factors levels activate in astrocytes the gene expression and regulate the transcription factors so that the subsets of progenitors are spontaneously engaged in different pathways of development [8]. During their differentiation, between the glial precursors and the microenvironment there is a mutual influence: cells secrete various soluble factors, and, on the other hand, the extracellular matrix (ECM) molecules (e.g. lectican and tenascins family) have the ability to stimulate or to inhibit cells proliferation, maturation and migration [9, 10]. Thus, in his study, Haas C. *et al.* in 2012, observed that by treating GRP *in vitro* with specific culture media, different astrocytic phenotypes were obtained (e.g. A2B5-/GFAP+ with a flat morphology fibroblast-like when treated with fetal bovine serum and A2B5+/GFAP+ star-shaped astrocytes when treated with both basic fibroblast growth factor (bFGF) and ciliary neurotrophic factor (CNTF) [8].

For example, if we consider a multipotent stem cell as a source of astrocytes, but initially, this cell has produced neuronal precursors, the turn towards glial differentiation implies a multi-step process. At first, a specific receptor on the surface of the multipotent stem cell modifies its structure to gain affinity for growth factors such as: fibroblast growth factor (FGF) and epidermal growth factor (EGF); then, the resulting glial precursor is subjected to the action of signalling molecules (e.g. CNTF, bone morphogenetic proteins (BMF) and EGF) that will control and continue its maturation [9, 10].

However, further research is needed in order to identify the heterogeneous subpopulations of astrocytes progenitors and accurately characterise them by new antigenic markers, physiological properties or molecular profiles [1].

At present, three distinct pools of glial progenitors have been described in the germinal niches of the cerebral cortex: a) radial cells of the ventricular zone b) postnatal glial progenitor cells of the subventricular zone and c) glial-restricted precursors (GRP) - also found in the embryonic spinal cord (see Table 1) [3, 8].

**Table 1:** **Ontogenetic astrocyte progenitor pools**

	Radial glia	Postnatal glial progenitor cells	Glial restricted precursors	
Origin	Neuroepithelial cells [1]	• Radial glia [11, 12]	Neuroepithelial cells skipping the radial glia stage [13, 14]	
		• Dlx2 (distal-less homeobox 2) [3]		
		• Local glial progenitors [1]		
Location	Ventricular zone [1]	• Subventricular zone	• Embryonic spinal cord [8]	• Optic nerve [8]
		• Dorso-lateral subventricular zone		
		• Marginal zone [1, 11, 12]		
Characteristics	Multipotential cells [11, 12]	• Multipotential cells	Tripotential cells [8]	• Bipotential cells O-2A, O-2A/OPC [8, 15]
		• Bipotential cells [3]		
Roles	• Progenitors for neurons and glial cells	• Intermediate progenitors for astrocytes and oligodendrocytes [3]	• Promote neuroprotection	• Tumor genesis (oligoastrocytomas, multiform glioblastomas) [15]
	• Guidance of neuronal migration [11, 12]		• Reduction of glial scar	
			• Formation and axonal growth [8]	
Type of resulting astrocytes	• Star shaped specialised cortical astrocytes	• Cortical astrocytes	• Self-renewal	• Astrocytes type 2 and oligodendrocytes (*in vitro*)
		• White matter astrocytes	• Astrocytes types 1, 2 and	
	• Bergmann glia in the cerebellum [3, 16]	• Oligodendrocytes [3]	• Oligodendrocytes [8]	• Oligodendrocytes (*in vivo*) [8, 15]

The grey matter protoplasmic astrocytes are mostly generated by embryonic radial glia but also from the intermediate progenitors arisen from neonatal subventricular zones. Due to their different origin, the two populations of astrocytes will display different patterns of gene expression, which will enable potential different functions. The white matter fibrous astrocytes originate, instead, mainly from neonatal subventricular zone progenitors [1].

### Astrocytes-like neural progenitors

An unexpected finding in the astrocyte research is the identification in the adult neurogenic zones - subventricular zone (SVZ) and subgranular zone (SGZ) - of a subtype of astrocytes considered to be the local stem cells. Regarded as mature astrocytes due to the expression of GFAP and glycogen granules, these cells unusually display features of both radial glia and neural progenitors (e.g. synaptic mediators’ release) [1].

It was demonstrated that the specific pro-neural genes (e.g. neurogenin-2 and Mash1) enable these astrocytes to regain their stem cells properties being able to differentiate into neurons [1]. Additionally, the embryonic extracellular matrix molecules present in the neurogenic niche are capable to maintain these cells’ “stemness” [1, 17].

In the adult SVZ and SGZ, two distinct population of neural progenitors (multipotent neural stem cells) express GFAP [1, 18–20]. The SVZ progenitors and give rise to neuroblasts which migrate to the olfactory bulb (to become olfactory interneurons) [1, 19–22]. GFAP-expressing cells found in the SVZ are also been referred to as astrocytes-like cells or B cells. From the histological point of view, these cells are irregular in shape, filling in the spaces between neighbouring cells; their cytoplasm is pale with few organelles (e.g. free ribosomes) but numerous intermediate filaments; the nuclei are also irregular due to the invaginations on their surface. There are significant differences between the two types of SVZ astrocytes. Type 1 (i.e. B1 cells) are larger, with euchromatic nuclei and are located in the proximity of the ependymal cells. Type 2 (i.e. B2 cells) are smaller, with hyperchromatic nuclei and are mostly adjacent to the striatal parenchyma. The SGZ neural progenitors generate newborn granular neurons [1].

Another type of stem cell which expresses GFAP can be found in the adult SVZ but it is not certain that these adult stem cells are, in fact, astrocytes. They have different molecular features, because they express nestin (an intermediate filament), that characterise only embryonic astrocytes, reactive astrocytes or neuroblasts and intermediate progenitors [1].

Considering the high plasticity of astrocytes, the GFAP expressing cells in the neurogenic niche can simultaneously behave as both astrocytic and neural stem cells [1].

### Astrocytic markers and stains

**Figure 1:**
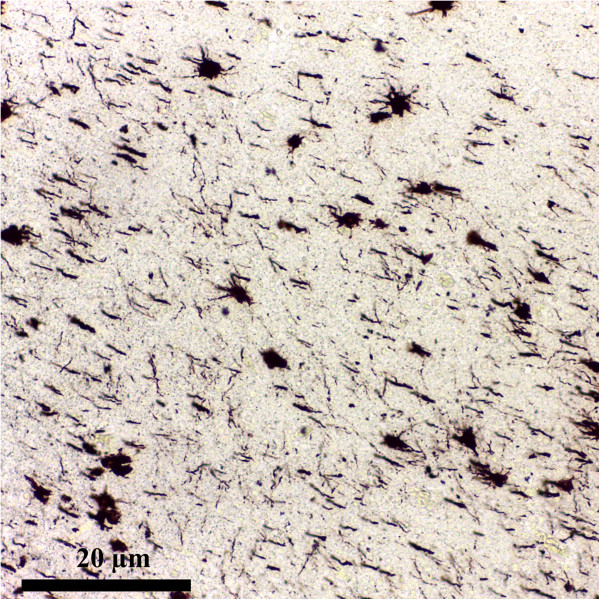
**Astrocytes overview.** Metalic impregnation Ramon Y Cajal Ob. 20x. Human brain (personal collection).

**Figure 2:**
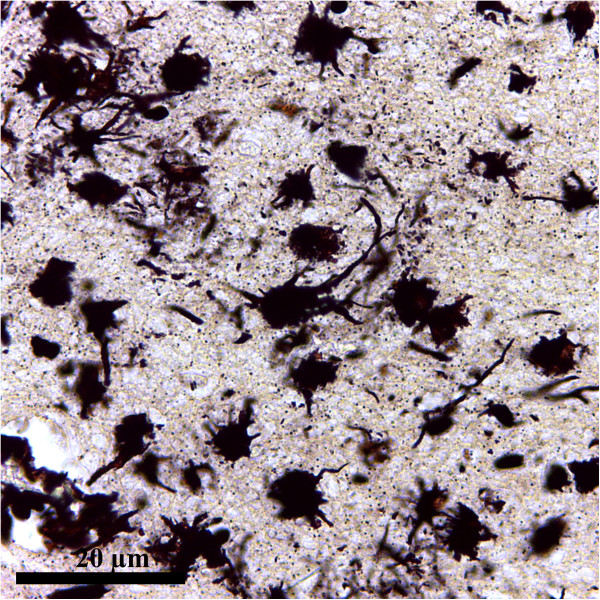
**Astrocytes overview.** Metalic impregnation Ramon Y Cajal Ob. 40x. Human brain (personal collection).

**Figure 3:**
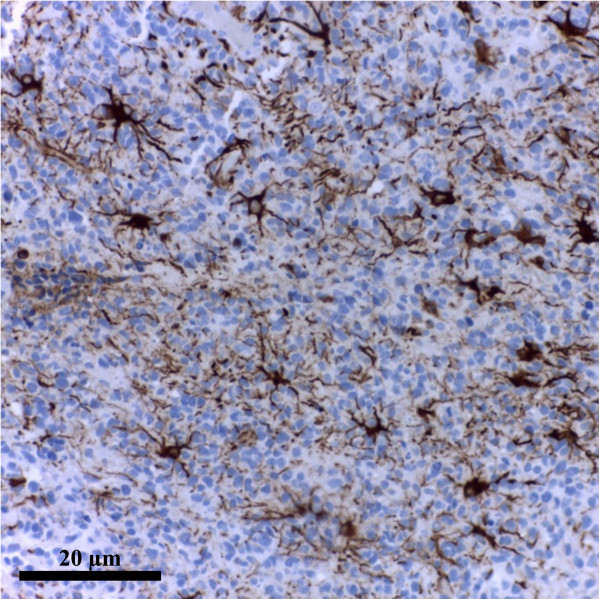
**Astrocytes overview.** GFAP Clone GF2 DAKO. Human brain. Ob. 20x. (personal collection).

Important advances in technologies to study the nervous tissue enabled the knowledge of astrocytes characteristics (see Table 2), Figures 1, 2 and 3. (*All images presented in here, are microphotographs of human brain samples prelevated by autopsy in compliance with the Protocol elaborated by the Ethics Committee of “Iuliu Hatieganu” University of Medicine and Pharmacy Cluj-Napoca).*

**Table 2:** **Astrocytic markers and stains**

Procedures	Characteristics	Results	Observation
*Hematoxylin and eosin stain (H-E)* [23]	Routine staining for basic morphology	Nuclear details	• Astrocytes are difficult to identify **(nuclei**: small, pale, ovoidal, euchromatic and centrally situated, are mimicking those of small neurons; **cytoplasm** and **cellular processes** are undifferentiated from those of neighbouring neurons)
		Cytoplasm extracellular protein components	• The occasionally pericellular hallo (autolitic modification) impose a differential diagnosis with the oligodendrocytes [23]
*Mallory’s (phosphotungstic acid – hematoxylin) stain* [24]	Special stain	Astrocyte processes (deep blue)	
*Orange-acridine stain* [24]	Special stain	Cellular body	• Reveals the astrocytic hyperplasia, without the modification of the cytoplasm aspects [24]
*Metallic impregnations* [23]		Nuclei	• Reveals the cellular characteristic star- shaped aspect
• *Del Rio Hortega method*	• Special technique with ammonia silver carbonate	Cytoplasm processes	• The abundant cytoplasm surrounding the nuclei differentiates the astrocytes from oligodendrocyte
• *Ramon y Cajal method* (see Figures 1 and 2)	• Special technique with gold chloride		• The fibrillar aspect of the cytoplasm is due to the material formed by the aggregation of GFAP intermediate filaments
• *Golgi stain*	• Special technique with silver nitrate		• The vascular endfeet are easy to identify.
			• Protoplasmic astrocytes, due to their proximity to the blood vessels, are able to contact the vessel directly by their cell body
			• The perivascular hallo is considered to be an artefact [23].
*Electron microscopy* [24]		Cytoplasm intermediate GFAP	• Cytoplasm pale , with lack of organelles
			• The clear, perivascular spaces indicate excessive dilatation of astrocytic processes due to water imbibitions
			• The ultrastructural resemblance between normal and well differentiated neoplastic astrocytes is one of the arguments against the use of this method for positive diagnosis of low grade glioma [24]
*Immunohistochemistry*			GFAP represents an integrator of the cellular space, but it is also implicated in complex cellular events, such as cytoskeleton reorganisation, myelination, cellular adhesion and several signalling pathways [23, 24].
• *GFAP* (intracytoplasmic protein, with 50 Kda molecular weight, considered the major component of glial fibrils and a marker of astrocytic differentiation) [23, 24] (see Figure 3)	• Golden standard for the definition of astrocytes	Cell body	• Fibrillary astrocytes contain a massive amount of GFAP in their cell bodies and processes unlike protoplasmic astrocyte.
	• There are different clones of antiGFAP antibodie, characteristic to the different research	Cell processes (positive immunostaining reaction: brown spots)	• Protoplasmic astrocytes are much larger than their GFAP-defined profiles due to the presence of numerous fine processes that are GFAP-negative
	• Laboratories (e.g. GF2 DAKO clone; Astro 1) [23, 24]		• In astrocytomas, along with the enhancement of malignity, the intracellular quantity of GFAP is progressively reduced; therefore the evaluation of GFAP immunohistochemical staining will enable the immunophenotypic characterisation of the investigated glial tumors and the confirmation of histopathological diagnosis
			• Not all the cells in the CNS that express GFAP are astrocytes (e.g: astrocyte-like cells from the SVZ-derived from radial glia, ependymal cells) [1, 25, 26]
			• GFAP has also been located in rat kidney glomeruli and peritubular fibroblasts [1, 27], Leydig cells of the testis [1, 28], skin keratinocytes [1, 29], osteocytes of bones, chondrocytes of epiglottis, bronchus [1, 30], and stellate-shaped cells of the pancreas and liver [1]
*S100B* (belongs to the S100 family of EF-band calcium binding proteins [1, 31]).	There are different clones of anti S100 antibodies, characteristic to the different research laboratories (e.g. MAB079, CBL410.)	Cell membrane	• Expressed by a subtype of mature astrocytes that ensheath blood vessels and by NG2-expressing astrocytes [1, 31]
*Other astrocytic markers*			
• *GLT-1* (the glutamate transporters GLAST) [6]			• GLT-1 is expressed by all astrocytes and provide punctuate staining [6]
• *Human EAAT2* (excitatory amino acids, 1 and 2 for human brain) [6]			
• *Glycogen granules*[6]		Cytoplasm	
• *Gglutamine synthase (GS)*[1, 32–35]	GS- enzyme that catalyzes the conversion of ammonia and glutamate to glutamine	Cytoplasm	GS is expressed also by oligodendrocytes [1, 32–35]
*Kir4.1* (inwardly rectifying K^+^ channels) [1, 36, 37]			Kir4.1 are only expressed by a subset of astrocytes [37]
• *Aquaporin 4 channels*[1, 38]		Cell processes	• *Aquaporin 4 channels* is localized in some parts of the astrocytic processes rendering identification of the whole cell difficult to interpret [38]
• *AldhL1* (aldehyde dehydrogenase 1 family, member L1) [1, 39].		Genome	• All astrocytes
*Battery of tests*[40]• GFAP-driven GFP (green fluorescent protein) expressionGFAPprotein expression, S100ß immunostaining	Combinatorial approach		• Nine different classes of astrocytes has been identified, that included Bergmann glia, ependymal glia, fibrous astrocytes, marginal glia, perivascular glia, protoplasmic astrocytes, radial glia, tanycytes and velate glia [3, 40]
• GFAP expression glutamate response [41]			• Define the phenotype of an astrocyte population as (GFAP^+^/NG2^-^; T^+^/R^-^) which is distinct from NG2-glia (GFAP^-^/NG2^+^ T^-^/R^+^) [41]
Dye-filling techniques [6, 42](e.g. sharp electrode, patch clamp recordings, single cell electroporation)	Special techniques that identify cells recorded *in situ* after filling them with a dye present in a micro-electrode	Cell body	• This technique has the advantage that the cells to be studied can be *preselected* in living tissue [6, 42]
	It is suplemented by use of presumed astrocyte-	Cell processes	• However, proteins and promoter activation are subjects to change. Hence one can have a GFAP(-) cell that one should call an astrocyte because it has these other properties [6, 42]
	Specific promoters to drive synthesis of fluorescent proteins		• Using these procedures the domain organisation of astrocytes has been demonstrated along with the fusiform morphology of astrocyte nucleus, both playing a possible role in pathology [3, 43, 44]
Transgenic techniques (use transgenic mice) [1]	Visualize fluorescent astrocytes	Cell body	• Mice specific for astrocytes express [1]
		Cell processes	- GFP
			- Enhanced GFP under the human GFAP promoter (hGFAP-GFP mice)
			- GLT-1-GFP
			- BLBP-dsRed2

For example, the grey matter protoplasmic astrocytes, are generated from embryonic radial glia and, to a lesser extent, from intermediate progenitors migrating from the neonatal subventricular zones. These two pathways of development will generate astrocytes with different patterns of gene expression and possibly different functions.

On the other hand, the white matter fibrous astrocytes are predominantly generated from neonatal subventricular zone progenitors [1].

Yet, it is important to recognize that subsets of progenitors will spontaneously differentiate in culture, as the intrinsic program of the cells modulates the process of cell division and differentiation together with culture conditions. Nevertheless, treatment of GRP cultures with fetal bovine serum (FBS) resulted in the production of A2B5-/GFAP + astrocytes with a fibroblastlike flat morphology, whereas exposure to basic fibroblast growth factor (bFGF) together with ciliary neurotrophic factor (CNTF) produced mostly process-bearing A2B5+/GFAP + astrocytes. Further research is needed to elucidate the identity of the different classes of intermediate progenitors or to obtain a clear antigenic signature of the lineage [8].

The development of astrocytes from a multipotent stem cell that prior to this has produced neuronal precursor cells, implies a specific differentiation via a multi-step process. The switch toward the glial differentiation is regulated by a change in receptor composition on the cell surface and responsiveness to fibroblast growth factor (FGF) and epidermal growth factor (EGF); futhermore, signaling molecules like CNTF, bone morphogenetic proteins (BMF), and EGF will continue to drive the glial precursor cell into the astroglial direction. However, the early astrocytes will interact with their microenvironment not only by releasing and responding to diverse soluble factors, but also expressing a wide range of extracellular matrix (ECM) molecules, as proteoglycans (lectican family) and tenascins. Lately it is considered that these ECM molecules have the ability to participate in glial development (e.g. the matrix protein Tenascin C (Tnc), proved to be an important regulator of astrocyte precursor cell proliferation, maturation and migration during spinal cord development) and those expressed by reactive astrocytes under pathophysiological conditions, are known to act mostly in an inhibitory fashion [9, 10].

### Astrocytes as a source of stem cells

The most recent and exciting finding in the astrocyte field, which challenges the traditional definition of astrocyte itself, is the discovery that there is a subclass of mature astrocytes which represent the stem cells in the adult neurogenic zones. The GFAP-expressing stem cells have characteristics of embryonic radial glia and mature astrocytes, but display subtle differences and retain properties of neural progenitors. These stem cells act in concert with resident astrocytes to contribute to cell genesis and maintaining the neurogenic environment, the niche. Perhaps these cells are retained in a transitional stage between radial glia and astrocytes, due to the persistence of embryonic extracellular matrix molecules. This permissive environment in the neurogenic niche allows the retention of intrinsic genetic programs to maintain “stemness” [1, 17]. It was shown that, the proneural genes *neurogenin-2* and *Mash1* possess the ability to reprogram these astrocytes to stem cells that can generate neurons [1].

In the adult subventricular zone (SVZ) and subgranular zone (SGZ), two distinct population of neural progenitors (multipotent neural stem cells) express GFAP [1, 18–20] and give rise to neuroblasts that either migrate to the olfactory bulb (to become olfactory interneurons) [1, 19, 21, 22] or generate newborn granule neurons. GFAP-expressing cells of the SVZ have been termed SVZ astrocytes, astrocyte-like cells or B cells. The histology of these cells comprises irregular contours that filled the spaces between neighbouring cells, irregular nuclei with invaginations, and light cytoplasm with few free ribosomes. They also expressed abundant intermediate filaments. Differences were found between the two types of astrocyte-like cells. Type B1 cells are larger than type B2 cells and possess euchromatic nuclei; they are adjacent to ependymal cells. Type B2 cells are smaller with hyperchromatic nuclei and are mostly located at the interface with the striatal parenchyma [1].

Another type of stem cell which expresses GFAP can be found in the adult SVZ but it is questionable whether these adult stem cells really belong to the astrocyte family. They has different molecular features, because they express nestin (an intermediate filament), that characterise only embryonic astrocytes, reactive astrocytes or neuroblastes and intermediate progenitors [1].

In conclusion, there is much need for further studies to be conducted in an attempt of finding new antigenic markers, physiological properties or molecular profiles for a better definition of these varieties of stem cells and to answer to challenging question as the ability of every astrocyte to revert to stem cells given the right environment [1].

### Astrocytic markers and stains

Many novel tools to study astrocytes were given by the technological advances over the past decades. From the early Golgi stains to immunostaining for glial fibrils, or the recent dye-filling techniques (e.g. sharp electrode, patch clamp recordings, single cell electroporation), and transgenic approaches to visualize fluorescent astrocytes, our understanding of astrocyte characteristics has dramatically evolved [1] (see Table 2), Figures 1, 2 and 3.

**Figure 4:**
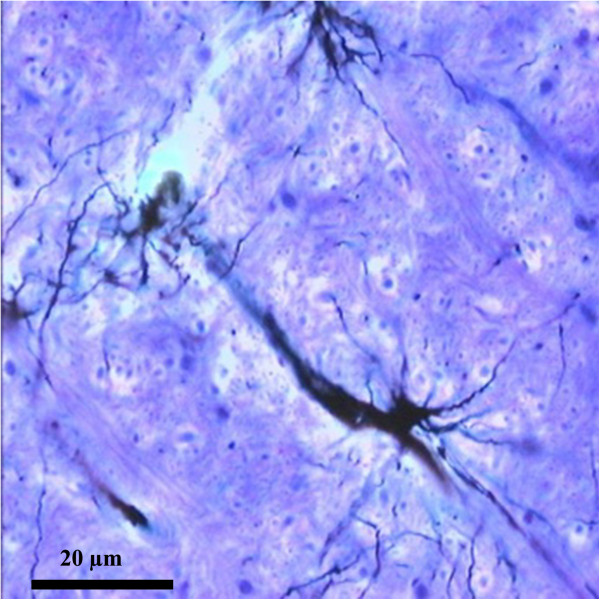
**Protoplasmic astrocyte proximal to a blood vessel.** Metallic impregnation Ramon Y Cajal. Ob. 20x. Human brain (personal collection).

The morphological features and the close relationships with both neurons and capillaries are the most constant characteristics that can be used to define the astrocytic phenotype [3] (see Figure 4).

### Types and morphology

**Figure 5:**
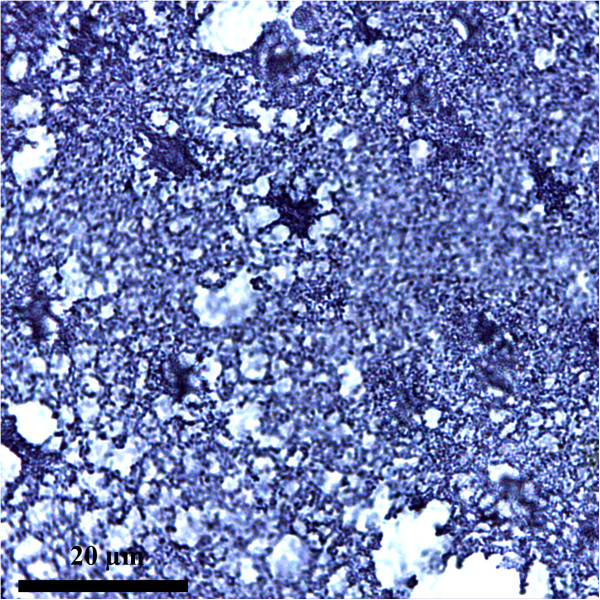
**Protoplasmic astrocyte.** Metallic impregnation Ramon Y Cajal Ob. 100 immersion. Human brain (personal collection).

**Figure 6:**
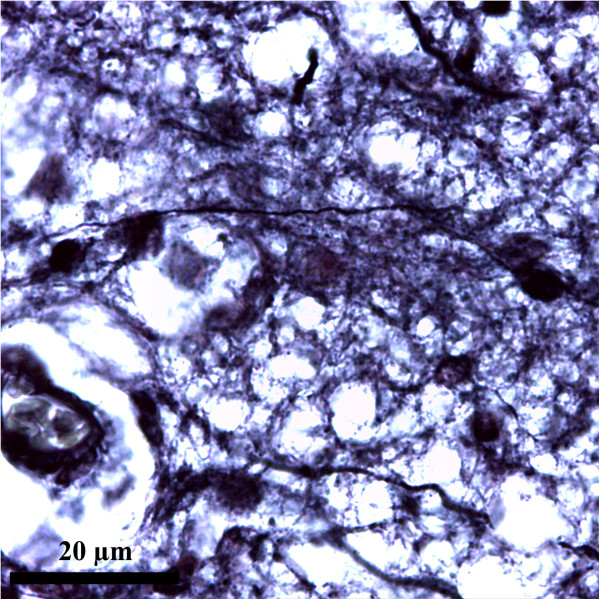
**Fibrous astrocyte.** Metallic impregnation Ramon Y Cajal Ob. 100 immersion. Human brain (personal collection).

Two major classes of astrocytes were first described in the 19th century by using the Golgi staining, which revealed their distinct morphological pattern: the protoplasmic and fibrous astrocytes. Nowadays the classification of astrocytes into fibrous and protoplasmic is considered to be outdated [45]; their morphological diversity can be illustrated by specialised classes of astrocytes represented by: the cerebellar Bergmann and Fananas glia, the Müller glia of the retina, the pituicytes of the neurohypophysis and the interstitial cells of the epiphysis. Additionally, in humans and primates two novel subtypes of astrocytes have been described: interlaminar astrocytes and varicose projection astrocytes [3, 4, 46–49]. (see Table 3) Figures 5 and 6.

**Table 3:** **Types of astrocytes**

Types of astrocytes	Location	Morphology	Functions	Particularities
***Protoplasmic astrocytes***	Uniformly distributed within the grey matter [3]	Bushy appearance, with numerous short, branched, thick processes [50]. The cell body is ovoid or fusiform (see Figure 5)	• Form the blood–brain barrier	Their processes exhibit endfeet enveloping the synapses and the blood vessels [51]. The processes express
			• Regulate the blood flow	
			• Neuronal metabolism	• Receptors for neurotransmitters, cytokines, growth factors
			• Implicated in the synapse function	• Transporters
			• Fluid, ion, pH and transmitter homeostasis [45]	• Ion channels [7]. In rodents, there is minimal overlapping between the processes of the neighbouring astrocytes [43, 44, 52–54]. In humans, the superposition of the domains occupied by the astrocytes processes is augmented [3]
***Fibrous astrocytes***	Within the white matter, oriented longitudinally, along the nervous fibers bundles [1]	Star-shaped cells. Posses long, thin and straight processes [45] (see Figure 6)		Their endfeet processes envelop the nodes of Ranvier and the blood vessels [45]
***Interlaminar astrocytes***	In the molecular 1st layer of the cerebral cortex, next to the pial surface	Spherical cell bodies and processes	Unknown Support the calcium wave propagation in humans [3]	Are found only in humans and primates. Their processes are included in the pial glial membrane, creating a thick network of GFAP fibers [46–49]
***Varicose projection astrocytes***	In the 5th and the 6th layers of the cerebral cortex	Exhibit 1 to 5 long processes (up to 1 mm in length), characterized by evenly (10 μm) spaced varicosities [3, 46]	Unknown	Were identified only in humans and chimpanzees. They are GFAP^+^ cells [3, 46]
***Bergmann glia (epithelial glial cells)***	In the Purkinje-cell and the granular layers of the cerebellar cortex	Posses long processes extending towards the molecular layer of the cerebellar cortex, in a fan-like arrangement, exhibiting pial vascular endfeet [23]	Implicated in synapse function: capable to interfere with synaptic transmission by communicating with neurons via the extracellular space, by modulating ion concentrations or transmitter levels in the synaptic cleft [23]	Display receptors with distinct biophysical and pharmacological features allowing them to sense the activity of synapses [23]
***Fananas cells***	In the molecular layer of the cerebellar cortex	Posses several short side processes with a characteristic feather-like arrangement [23]		
***Müller cells***	In the 6th layer of the visual retina		Supportive cells: they form the inner and the outer limiting membranes	The limiting membranes consist of junctional complexes between the cellular processes of the Müller cells
			The outer membrane separates the external segment of the photoreceptor cells from the cell bodies and the outer membrane separates the retina from the vitrous body [23]	They have an intense metabolic activity and contain microfilaments and glycogen within their cytoplasm [23]
***Pituicytes***	In the neurohypophysis	Irregular in shape with many cytoplasmic processes extending in the proximity of the capillaries and surrounding the Herring bodies [24]		Their cytoplasm contains lipid droplets and pigment granules.
				They are immunoreactive for GFAP, vimentin and S100 protein [24]
***Inerstitial epiphysial cells***	In the epiphysis	Exhibit cytoplasmic processes		Contain numerous filaments within their processes [23]

The above presented heterogeneity of astrocytes could arise from separate lineages, plasticity of mature cells (motility and reactivity after injuries), or association of both factors [3, 54]. Methods of molecular biology, like time-lapse studies in slice culture, demonstrated the participation of astrocytes in synaptic remodelling, since the astrocytic processes are motile and enwrap active synapses [3, 55, 56].

It is well-known that mature astrocytes can exhibit forms of plasticity: motility and reactivity after injuries. Time lapse studies of astrocytes in acute slice and slice culture have shown that astrocyte processes act much like dendritic spines; they are frequently motile and contact active synapses [3, 55, 57], the role of this feature implying the synaptic remodelling.

### Reactive astrocytes

Astrocytes become reactive notably after injuries, when the intermediate filament proteins (e.g. GFAP, vimentin, nestin) are upregulated, becoming larger and there is an alteration of the domain organization [58, 59].

The reactive morphological variants comprise two main categories: the individualised and the global reactive astrocytes. Individualized reactive astrocytes encompass several types: pilocytic astrocyte, gemistocytic astrocyte, type I and II Alzheimer astrocytes. The global reactive astrocytes are the characteristic feature of reactive astrogliosis (see Table 4) [60].

**Table 4:** **Individualized reactive astrocytes variants**

Individualized reactive astrocytes variants	Causes	Morphology	Particularities
Pilocytic astrocytes [23, 24]	• In mild and moderate injuries as individual form of reactive astrocytes	• Elongated, bipolar cell body	These cells contain the Rosenthal fibers (specific but inconstant eosinophilic, cork-screw shaped elements), representing an advanced stage of cellular degeneration in astrocytoma
	• Astrocytoma	• Fusiform nuclei	
		• Thin and long hair-like GFAP^+^ processes	
Gemistocytic astrocytes [23, 24]	• In mild and moderate injuries as individual form of reactive astrocytes	• Large, dilatated, oval cell body	The organelles are numerous and located in the central zone of the cell body. The glial filaments are also numerous and peripherally arranged, beneath the plasmalemma
	• In gemistocytic astrocytoma as a characteristic feature of this tumors [23]	• Few thick cytoplasmic processes	
		• Abundant, deeply eosinophilic cytoplasm	
		• Polymorphic nuclei, frequently eccentrical.	
Alzheimer type I astrocytes [23, 24]	• Progressive multifocal leuco-encephalopathy	• Enlarged cell body	
		• Numerous nuclei	
Alzheimer type II astrocytes [23, 24]	• Associated with high blood ammonia in hepatic encephalopathy	• Enlarged cell body	Ammonia taken up by astrocytes is converted to osmotically active glutamine, resulting in astrocytic swelling
	• In Wilson disease	• Vesicular nuclei with one or more nucleoli	

*Reactive astrogliosis*, a hallmark of all forms of CNS injuries, is the result of a multi-step process involving gradates changes in astrocytes.

Histopathological examinations of human brain in various neurological conditions have provided different degrees of reactive astrogliosis. According to Sofroniew et al., the following categories of reactive astrogliosis can be identified: mild to moderate astrogliosis, severe astrogliosis and the glial scar [60].

Mild to moderate astrogliosis is a manifestation of various disorders (systemic viral and bacterial infections, non-penetrating trauma) and also found in the distant areas surrounding the focal cerebral lesions [60]. The changes associated with mild to moderate astrogliosis are reversible if the triggering mechanism has resolved. In this type of injuries, subtle alterations occur in the expression of molecules implicated in the cellular activity: cell structure, energy metabolism, intracellular signaling, membrane transporters and pumps [60].

Various functional categories of genes and molecules modulated by reactive astrocytes can be either upregulated or downregulated, depending on the trigger or the moment after the insult. As a result of these alterations, specific histopathological features can be identified in astrocytes (see Table 5).

**Table 5:** **Reactive astrogliosis**

Reactive astrogliois	Changes in astrocytes morphology	Changes in molecules expression	
		Upregulated molecules	Upregulated or downregulated molecules
Mild to moderate astrogliosis	• Hypertrophy of cell body	• Structural elements: GFAP, nestin, vimentin	• Inflammatory cell regulators: cytokines, growth factors, glutathione
	• Astrocytes processes are are numerous and thicker	• Transcriptional regulators: STAT3, NFκB, Rheb-m TOR, cAMP, Olig2, SOX9 [61–65].	• Transporters and pumps: AQP4 and Na^+^/K^+^ transporters [61, 66–69]
			• Glutamate transporter [70–73]
	• The non-overlapping domains of individual astrocytes are preserved		• Vascular regulators: PGE, NO [74, 75]
			• Energy provision: lactate [76]
			• Molecules implicated in synapse formation and
Severe astrogliosis and glial scar	• Intense hypertrophy of cell body		• Remodeling: thrombospondin and Complement C1q [77, 78]
	• Significant extension of processes		• Molecules implicated in oxidative stress and providing protection from oxidative stress: NO, NOS, SOD, Glutathione [67, 68, 79]
	• Proliferation		
	• Overlapping of individual domains		
	• Substantial reorganization of tissue architecture [60]		

Severe diffuse reactive astrogliosis is characterised by permanent, pronounced and long-lasting changes and it is found in the areas surrounding severe focal lesions or infections, as well as in neurodegeneration [60].

Compact scar formation occurs in most severe injuries, such as overt tissue damage (e.g. penetrating or contusive trauma), inflammation initiated by invasive infections or abscesses, neoplasm and chronic neurodegeneration. Reactive astrogliosis reaches its highest level of activation: astrocytes undergo intense proliferation, and their long, branched processes overlap (see Table 5) [60].

Any cell type in the CNS (e.g. neurons, all types of glial cells, endothelial cells and leucocytes) is potentially able to release the molecular mediators of astrogliosis [60]. Signaling pathways and molecules implicated in mediating specific aspects of reactive astrogliosis include: STAT3 (signal transducer and activator of transcription 3), NFκB (nuclear factor kappa B), cAMP (cyclic adenosine monophosphate), all these inducing upregulation of structural molecules (e.g. GFAP, vimentin, nestin) [62, 63, 80]; moreover, STAT3 induces astrocyte hypertrophy, scar formation and exerts anti-inflammatory effect [62]; NFκB exerts pro-inflammatory effect [63, 81]; Olig2, Endothelin-1 induce astrocyte proliferation [65, 82].

The newly formed cells in the glial scar derive from different sources: mature astrocytes that re-enter the cell cycle, NG2 progenitors and ependymal cells progenitors.

Molecular mediators and triggers leading to proliferation include: cytokines and growth factors (e.g. IL (interleukin) 6, LIF (leukemia inhibitory factor), CNTF, IL1, IL10, TGFβ (transforming growth factor), TNFα (tumor necrosis factor), INFγ (interferon), [63, 69]), Toll-like receptor ligants [83], LPS (lipopolysaccharide), molecules of oxidative stress (e.g. NO (nitric oxide) and ROS (reactive oxygen species) [67]), modulators and neurotransmitters (e.g. noradrenalin and glutamate [84]), ischemia associated hypoxia and glucose deprivation [67], neurodegeneration associated amyloid- beta [85] and ATP (adenosine triphosphate) released by cell injury [86].

Reactive astrocytes interact with other cell types: fibromeningeal cells and NG-2 positive glia and are associated with a dense collagenous extracellular matrix to form complex glial scars [60, 62, 87]. The changes leading to scar formation persist even after the triggering factor has been removed [61, 62, 88, 89].

Recent evidence suggest that the glial scars might have a beneficial role, as they form narrow, dense and compact barriers; these barriers delimit the periphery of severe tissue damage, isolate the lesion, thus preventing inflammatory cells and infectious agents from spreading into the healthy parenchyma [90].

## Conclusions

This part of the review is an insight into the morphology and biology of astrocytes, with an emphasis on the latest findings concerning the novel cell subtypes, the developmental lineages and their functions.

From the very first description of astrocytes in the 19^th^ century, these cells concept has been at a standstill until recently when it remarkably progressed.

The variety of glial precursors, their ability to display stem cells features and different adult astrocytes morphology certify that the astrocytic phenotype is influenced by both the local cytoarchitecture and the functional requirements in specific brain areas.

## Abbreviations

AldhL1: Aldehyde dehydrogenase 1 family, member l1
AQP4: Aquaporin 4
ATP: Adenosine triphosphate
BBB: Blood–brain barrier
bFGF: Basic fibroblast growth factor
BLBP: Brain lipid basic protein
BMF: Bone Morphogenetic Proteins
cAMP: Cyclic adenosine monophosphate
CNS: Central nervous system
CNTF: Ciliary neurotrophic factor
Dlx2: Distal-less homeobox 2
ECM: Extracellular matrix
EGF: Epidermal growth factor
EAAT: Excitatory amino-acid transporters
FBS: Fetal bovine serum
FGF: Fibroblast growth factor
GLAST: Glutamate amino acid transporters
GLT: Glutamate transporters
GFAP: Glial fibrillary acidic protein
GFP: Green fluorescent protein
GRP: Glial-restricted precursors
HBB: Hemolymph-brain barrier
IL: Interleukin
INF: Interferon
Kir 4.1: Inwardly rectifying K + channels
LIF: Leukemia inhibitory factor
LPS: Lipopolysaccharide
NFκB: Nuclear factor kappa B
NO: Nitric oxide
NOS: Nitric oxide synthase
OLIG2: Oligodendrocyte transcription factor
PGE: Prostaglandin E
Rheb-m TOR: Ras homolog enriched in brain – mamalian target of rapamycin
ROS: Reactive oxygen species
SOD: Superoxide dismutase
SOX9: Transcription factor SOX9
STAT3: Signal transducer and activator of transcription 3
SGZ: Subgranular zone
SVZ: Subventricular zone
TGF: Transforming growth factor
Tnc: Tenascin C
TNF: Tumor necrosis factor
VZ: Ventricular zone.
